# Disruption of Astrocyte STAT3 Signaling Decreases Mitochondrial Function and Increases Oxidative Stress *In Vitro*

**DOI:** 10.1371/journal.pone.0009532

**Published:** 2010-03-10

**Authors:** Theodore A. Sarafian, Cindy Montes, Tetsuya Imura, Jingwei Qi, Giovanni Coppola, Daniel H. Geschwind, Michael V. Sofroniew

**Affiliations:** 1 Department of Neurobiology, David Geffen School of Medicine, University of California Los Angeles, Los Angeles, California, United States of America; 2 Department of Neurology, The Semel Institute for Neuroscience and Human Behavior, David Geffen School of Medicine, University of California Los Angeles, California, United States of America; University of North Dakota, United States of America

## Abstract

**Background:**

Astrocytes exert a wide variety of functions in health and disease and respond to a wide range of signaling pathways, including members of the Janus-kinase signal transducers and activators of transcription (Jak-STAT) family. We have recently shown that STAT3 is an important regulator of astrocyte reactivity after spinal cord injury *in vivo* [1].

**Methodology/Principal Findings:**

Here, we used both a conditional gene deletion strategy that targets the deletion of STAT3 selectively to astrocytes (STAT3-CKO), and a pharmacological inhibitor of JAK-2, AG490, in cultured astrocytes *in vitro*, to investigate potential functions and molecules influenced by STAT3 signaling in relation to mitochondrial function and oxidative stress. Our findings show that the absence of STAT3 signaling in astrocytes leads to (i) increased production of superoxide anion and other reactive oxygen species and decreased level of glutathione, (ii) decreased mitochondrial membrane potential and decreased ATP production, and (iii) decreased rate of cell proliferation. Many of the differences observed in STAT3-CKO astrocytes were distinctly altered by exposure to rotenone, suggesting a role for complex I of the mitochondrial electron transport chain. Gene expression microarray studies identified numerous changes in STAT3-CKO cells that may have contributed to the identified deficits in cell function.

**Conclusions/Significance:**

Taken together, these STAT3-dependent alterations in cell function and gene expression have relevance to both reactive gliosis and to the support and protection of surrounding cells in neural tissue.

## Introduction

Astrocytes play many essential roles in the healthy central nervous system (CNS), including regulation of blood flow, provision of energy metabolites to neurons, participation in synaptic function and plasticity, and maintenance of the extracellular balance of ions, fluids and transmitters [2], [3]. In addition, astrocytes are primary responders to CNS insults such as infection, trauma, ischemia and neurodegenerative disease, where they exert important tissue defense mechanisms or where their dysfunction may be involved in disease pathology [4]–[7]. Astrocytes are able to take part in this broad range of activities in part by being able to respond to a plethora of extra- and intra-cellular signaling mechanisms that regulate their functions and molecular expression in a context-dependent manner [8], [9]. Defining the signaling mechanisms that regulate astrocyte activities is of interest in understanding normal function in the healthy CNS, understanding disease mechanisms and identifying potential novel therapeutic targets.

Many molecules have been implicated as triggers of astrogliosis, including a broad group of growth factors and cytokines that signal through members of the Janus-kinase signal transducers and activators of transcription (Jak-STAT) signaling family [1], [10], [11]. Using a transgenic conditional gene deletion strategy, we have recently shown that one intracellular member of this family, STAT3, is a particularly important regulator of astrocyte reactivity after spinal cord injury *in vivo*, such that conditional deletion of STAT3 signaling from astrocytes attenuated reactive astrogliosis and disrupted scar formation, which was associated with increased inflammation, increased lesion size and decreased recovery of motor function [1]. In this study, we used both a genetic conditional deletion strategy and pharmacological inhibition of STAT3 to assess whether STAT3 deficient astrocytes have impaired function that may be detrimental to the astrocytes and ultimately to surrounding CNS tissue.

## Materials and Methods

### Materials

Generation of mice deficient in STAT3 expression selectively in astrocytes (STAT3-CKO) using glial fibrillary acidic protein (GFAP) promoter-directed Cre/loxP technology was described previously [1]. Mice were genotyped for Cre recombinase (Cre) and loxP sequence by DNA isolation from liver sections using the Qiagen DNAeasy kit (Valencia, CA) followed by PCR and agarose gel electrophoresis as described previously. Experiments were performed according to protocols approved by the Chancellor's Animal Research Committee of the Office for Protection of Research Subjects at the University of California, Los Angeles. Culture media and trypsin were obtained from HyClone (Logan, UT). Versene was purchased from Gibco (Gaithersburg, MD). Fluorescent probes dichlorofluoresceine-diacetate (DCF), dihydroethidine (HE), 5,5′,6,6′- tetrachloro-1,1′,3,3′-tetraethylbenzimidazolylcarbocyanin iodide (JC-1), MitoSOX Red, MitoTracker Green, monochlorobimane (MCB), and propidium iodide (PI) were obtained from Molecular Probes (Eugene, OR). The Cell-Titre-Glo luminescence viability assay kit was from Promega (Madison, WI). Primary antibody to GFAP was from Dako (rabbit, Carpenteria, CA), to tyrosine705-phosphorylated STAT3 (pSTAT3) was from Cell Signaling Technology, Inc. (rabbit 1∶500, Danvers, MA) to S100 was from QED Bioscience, Inc. (sheep, San Diego, CA), to p-p38, pJNK, and pErk MAP kinases were from Santa Cruz Biotechnology (rabbit 1∶1000, Santa Cruz, CA) and to actin and glutamine synthetase were from Sigma (rabbit 1∶4000, St. Louis, MO). Conjugated secondary antibodies to rabbit, goat, and donkey IgG were from Bio Rad (1∶1000, Hercules, CA). All other reagents were from Sigma.

### Cell Culture

Astrocyte cell cultures were prepared from 2-3-day-old STAT3 conditional knock-out (STAT3-CKO) mice and from littermate CRE-negative controls (STAT3 +/+) by a modification of the procedure of McCarthy and De Vellis [12]. Cerebral cortices from individual mice were isolated and, after removal of meninges, placed in 10×14 cm Stomacher bags with 2 ml culture medium (DMEM/Ham's F12 with 4.5 g/l glucose, 15 mM HEPES, 2 mM Glutamine, 10% fetal bovine serum and 1% Penicillin/Streptomycin). The tissue was disaggregated manually by finger compression for 3 min, triturated with 12 passes through a 1 ml pipetter and filtered through a 100 µm nylon cell strainer. Cells were then centrifuged at 800 rpm for 5 min, resuspended in culture medium and transferred to one T-75 culture flask. Media were replaced twice weekly. Upon reaching 90% confluence, cells were passaged (1∶3 in surface area) in order to generate sufficient cell number. For passage, cells were washed three times with phosphate-buffered saline (PBS) and incubated with 4 ml Versene (0.02% EDTA in PBS) for 20 min at 37° C. Then 1.5 ml of 0.05% trypsin and 0.02% EDTA were added and incubated 8 min at 37° C. Following addition of 1.5 ml trypsin neutralizing solution (Clonetics/Lonza), cells were collected by centrifugation (800g, 4 min) and counted with a hemocytometer.

Upon reaching 70–90% confluence, cells cultured in T75 flasks were used for gene expression array studies and in multi-well plates for all other experiments. Levels of GFAP and STAT3 expression did not change significantly in STAT3 +/+ cells during the culture periods used in these studies. Comparisons between STAT3 +/+ and STAT3-CKO for glutathione and ROS levels did not change as a function of days *in vitro* or between passages 2 and 3.

### Immunocytochemistry

Astrocyte cultures were prepared in 48-well plates and fixed with formalin. Cells were stained with anti-GFAP (1∶2000), anti S100β (1∶1000) or anti BrdU (1∶6000) followed by AlexaFluor-tagged secondary antibodies Alexa 488 (green) or Alexa 568 (red). Cells stained for BrdU were pretreated with 2M HCl for 30 min and rinsed three times with PBS. Images were recorded by fluorescence microscopy (Zeiss, Oberkochen, Germany).

### Western Blot Staining

Cells cultured in 12-well plates were washed three times with PBS and extracted with RIPA lysis buffer containing 0.8 µM aprotinin, 20 µM leupeptin, 10 µM pepstatin A, 2 mM phenylmethylsulfonyl fluoride, 20 mM NaF and 1 mM sodium orthovanadate. Following protein measurement using The Bio-Rad DC protein assay, 25 µg protein per well was applied to 4–12% gradient SDS polyacrylamide gels (Invitrogen). Electrophoresis was run at 100–130V for 2.5 hr followed by transfer to Hybond-P membranes (Amersham Biosciences) for 1.5 hr at 30V. Membranes were then blocked with 5% dry milk powder in Tris-buffered saline with 0.05% Tween 20 and stained with 1∶500 dilutions of various antibodies. Following secondary antibody staining, membranes were exposed to ECL chemiluminescent reagent (Amersham Biosciences - GE Health Care Bio-Sciences, Piscataway, NJ) and exposed to Kodak XAR5 film.

### Hydroethidine Assay for Superoxide

Cells in 96-well plates were washed with 200 µl of Krebs Ringer buffer (KR: 25 mM HEPES, pH 7.4, 125 mM NaCl, 5 mM KCl, 1.2 mM KH_2_PO_4_, 5 mM NaHCO_3_, 6 mM glucose, 1.2 mM MgSO_4_ and 1 mM CaCl_2_). Rotenone, or carbonyl cyanide p-trifluoromethoxy phenylhydrazone (FCCP) or DMSO vehicle (0.1%) was added in KR to the appropriate rows [13]–[16]. In select wells PBS was used to produce glucose-starvation. The plate was incubated for 30 minutes whereupon toxins were removed and wells washed once with 200 µl of Krebs Ringer. 10 µM HE in KR or PBS was then applied and fluorescence was read at Ex = 530, Em = 595 over a 30 minute period.

### Measurement of Reactive Oxygen Species, Glutathione, and Total Cell Number

Analysis of astrocyte reactive oxygen species (ROS) production was performed by a modification of previously described procedures [17]. Cells were washed twice with 200 µl of Krebs Ringer buffer KR. 20 µg/ml DCF-DA was added to the wells in 100µl of KR and the plate was sealed with mylar tape for 20 minutes. The plate was then washed twice more with 200 µl and 100 µl of KR and toxins added in 100 µl KR. Fluorescence readings were taken every 15 minutes for 1 hour at Ex = 485 and Em = 530. Then KR containing the toxins was removed and replaced with 100 µl 40 µM MCB in KR to determine glutathione (GSH) levels. The cells were incubated with the MCB for 20 minutes at 37°C and fluorescence readings taken at Ex = 390, Em = 460. Ten µl of 0.5 mM PI was added to the wells and, after incubation for 15 minutes at room temperature, red fluorescence was read at Ex = 535, Em = 590. Ten µl of 1.6 mM digitonin was then added to each well and incubated for 20 minutes at room temperature. PI fluorescence measurement was repeated to quantify total cell number which was used to normalize ROS and GSH levels. For each probe used, subtracted background values were obtained from wells containing fluorescent probe without cells.

### Mitochondrial Membrane Potential, ROS, and Mass

Mitochondrial membrane potential was measured by using the fluorescent probe JC-1 as described previously [18]. Rotenone, antimycin A, or FCCP were added to wells in a 96-well plate. Then 1 µg/ml JC-1 in culture media was added and the plate was incubated at 37° in a CO_2_ incubator for 1 hour. Red and green fluorescence measurements were taken at 2, 30, and 60 minutes using Ex = 485, Em = 530 for green and Ex = 530, Em = 590 for red. Following subtraction of blank values, red/green fluorescence ratios were calculated for each well using data from 60 min incubation. These data were compared with those from 2 and 30 min to verify appropriate time-dependent changes.

Mitochondrial-specific ROS was measured using MitoSOX Red. Astrocytes cultured in 96-well plates were exposed to 4 µM MitoSOX Red in culture media containing toxins or DMSO vehicle control. In order to measure total mitochondrial mass, parallel wells contained 0.1 µM MitoTracker Green which produces a green fluorescence independent of mitochondrial membrane potential. Following 2 hr incubation in a CO_2_ incubator, green and red fluorescence was measured as described for JC-1. Background fluorescence was determined from wells containing probes without cells and subtracted from respective red and green fluorescence values. Red fluorescence values were normalized to mitochondrial mass represented by averaged MitoTracker Green fluorescence values from respective STAT3 +/+ or CKO cells.

### ATP Assay

STAT3 +/+ and STAT3-CKO cells were grown in 96-well plates to 90% confluence. Media was removed from the wells and 50 µl of firefly extract from the CellTitre-Glo Viabiltiy assay kit (Promega) was added to each well. The plate was shaken for 3 minutes and samples were transferred to a white 96-well plate. The wells were rinsed with 50 µl of 10 mM Tris-HCl pH 7.4 and the rinse was added to the samples in the white plate. ATP standards from 2 to 400 pmole were used to generate a standard curve. Luminescence was measured with a Molecular Devices Spectra Max Gemini EM plate reader in top-read mode using SoftMax Pro v5 Software.

### Protein Assay

Cells in 96-well were washed three times with 200 µl KR. 50 µl of 1M NaOH were added to each well and the plate shaken for 3 minutes. 50 µl of 1M HCl was added to each well. Each well was mixed well with a pipette before 20 µl in duplicate were taken from each well and placed in a new 96-well plate. IgG was used as protein standard which included 20 µl of 1 M NaOH/HCl mix (1∶1). 20 µl of Bio-Rad Coomassie Blue reagent was added to standards and samples. The plate was read on the SLT Spectra plate reader at 620nm wavelength.

### Cell Proliferation Assay

Astrocytes cultured to passage 2 over a period of 3 weeks were plated into 96-well culture plates at a density of 5×10^3^/well. N-acetylcysteine (0.5 mM) or 1 mM deferroxamine mesylate in sterile H_2_O were added 1, 3, and 5 days after plating. After 1, 4, and 7 days in culture, cell number was quantified using propidium iodide in the presence of digitonin as described above. Values obtained after 1 day were subtracted from values after 7 days to derive the relative increase in cell number. GSH levels were assayed with monchlorobimane after 4 days as described above.

### Gene Expression Micro Array Studies

Total RNA was isolated from astrocytes cultured in T-75 flasks (P3, 70–80% confluence) using the Qiagen RNeasy Kit protocol for adherent cells. RNA yields ranged from 1 to 13 µg and had an A260/280 ratio >1.75. Four replicates were run per condition, for a total of 8 arrays. RNA quantity was assessed with Nanodrop (Nanodrop Technologies) and quality with the Agilent Bioanalyzer (Agilent Technologies). Total RNA (200 ng) was amplified, biotinylated and hybridized on Illumina Mouse Mouseref-8 Expression Beadchips v1.1, querying the expression of ∼22,000 Refseq transcripts, as per manufacturer's protocol. Slides were scanned using Illumina BeadStation and signal extracted using Illumina BeadStudio software (Illumina, San Diego CA). Raw data was analyzed using Bioconductor packages (www.bioconductor.org, [19]). Low level quality-control analysis was performed using several indices, including inter-array Pearson correlation, clustering based on variance, and the mean absolute deviation (MAD) using the top 1000 most variant probes [19]. Data were normalized using quantile normalization. Analysis of differential expression was performed using a linear model fitting (LIMMA package, [20]). After linear model fitting, a Bayesian estimate of differential expression was calculated and the threshold for statistical significance was set at p<0.005 (Bayesian modified t-test). Differentially expressed genes were classified according to gene ontology, using Bioconductor packages and online tools (DAVID/EASE, http://david.abcc.ncifcrf.gov/, WebGestalt, http://genereg.ornl.gov/webgestalt/). In DAVID, levels 3 and 4 of molecular function, biological process and cellular localization were selected. Literature data mining for co-occurrence of gene names and keywords of interest (e.g., oxidative stress, mitochondria etc.) was performed using Chilibot (www.chilibot.net). Pathway analysis was carried out using Ingenuity Pathway Analysis (Ingenuity Systems, www.ingenuity.com).

### Statistical Analysis

Data from in vitro cellular assays were analyzed by 2-way ANOVA with Bonferroni post-hoc test using Prism Graphpad software. Cell proliferation assays were analyzed by 1-way ANOVA with Tukey's post hoc test.

## Results

### Characterization of STAT3-CKO Astrocyte Cell Cultures

We have previously demonstrated the specificity of the STAT3-CKO transgenic model for targeting STAT3 gene deletion to astrocytes by (i) analyzing Cre mediated activation of reporter gene expression at the single cell level *in vivo* and (ii) analyzing the selective deletion of STAT3 and pSTAT3 from astrocytes *in vivo* and *in vitro* [1]. For the present study, we confirmed and extended these observations by characterizing in various ways primary astrocyte cultures prepared from STAT3-CKO mice.

**Figure 1:**
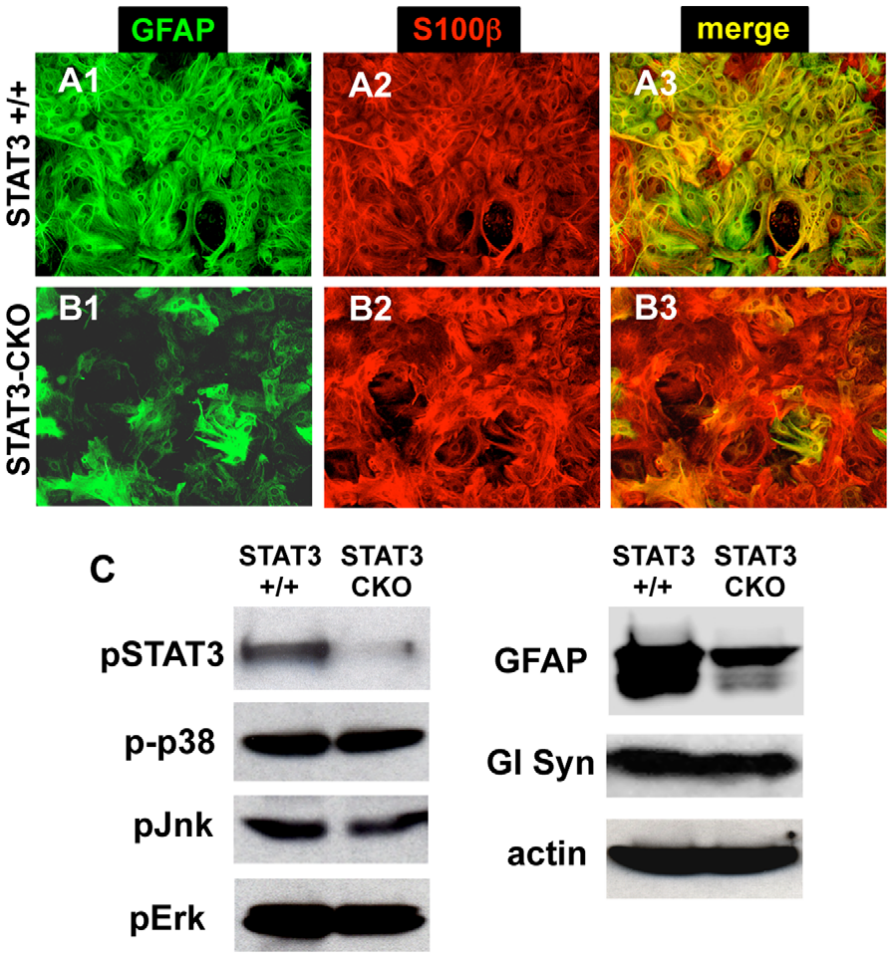
Immunofluorescence and Western blot staining of enriched astrocyte cell cultures derived from neonatal forebrain. (**A,B**) Single channel and merged images of double labeling immunofluorescence show that in control cultures (**A1–A3**) essentially all astrocytes express both GFAP and S100β, whereas in STAT3-CKO cultures (**B1–B3**) most astrocytes do not express detectable levels of GFAP but do express S100β. (**C**) Western blotting of primary astrocyte cultures shows markedly reduced expression of pSTAT3 and GFAP, but not of phosphorylated MAP kinases or glutamine synthetase (Gl Syn), in STAT3-CKO cultures as compared with controls. Equivalent amounts of total protein were applied to each lane.

To evaluate the effects of STAT3-CKO on the appearance and various molecular expression profiles of astrocytes *in vitro*, we used primary astrocyte cultures that are over 95% GFAP-expressing cells [21], [22]. Primary astroctye cell cultures prepared from perinatal STAT3-CKO mice grew well under standard conditions and had an appearance under phase-contrast microscopy similar to that of cells from littermate control mice negative for Cre expression. STAT3-CKO astrocytes exhibited a moderately reduced expression of GFAP, but expressed S100β or glutamine synthetase at normal levels as detected by immunocytochemistry (Figs. 1A,B) and Western blotting (Fig. 1C). In agreement with our previous report [1], STAT3-CKO cultures exhibited almost no detectable pSTAT3, whereas pSTAT3 was present in control (STAT3 +/+) cultures (Fig. 1C). These findings demonstrate that (i) STAT3 is activated and signaling in STAT3 +/+ astrocytes under basal culture conditions that contain serum, and (ii) our Cre-loxP model for STAT3-CKO effectively deleted signaling via STAT3. We also looked for potential effects of STAT3-CKO on other signaling pathways, and found no detectable differences in the levels of p-p38, pErk and pJnk MAP kinases between control and STAT3-CKO astrocytes (Fig. 1C). We therefore focused the present studies on the effects of STAT3-CKO on astrocyte functions under standard culture conditions in serum that are associated with a basal constitutive activation of STAT3.

### STAT3-CKO Alters Functions Related to Oxidative Stress and Antioxidant Defense

**Figure 2:**
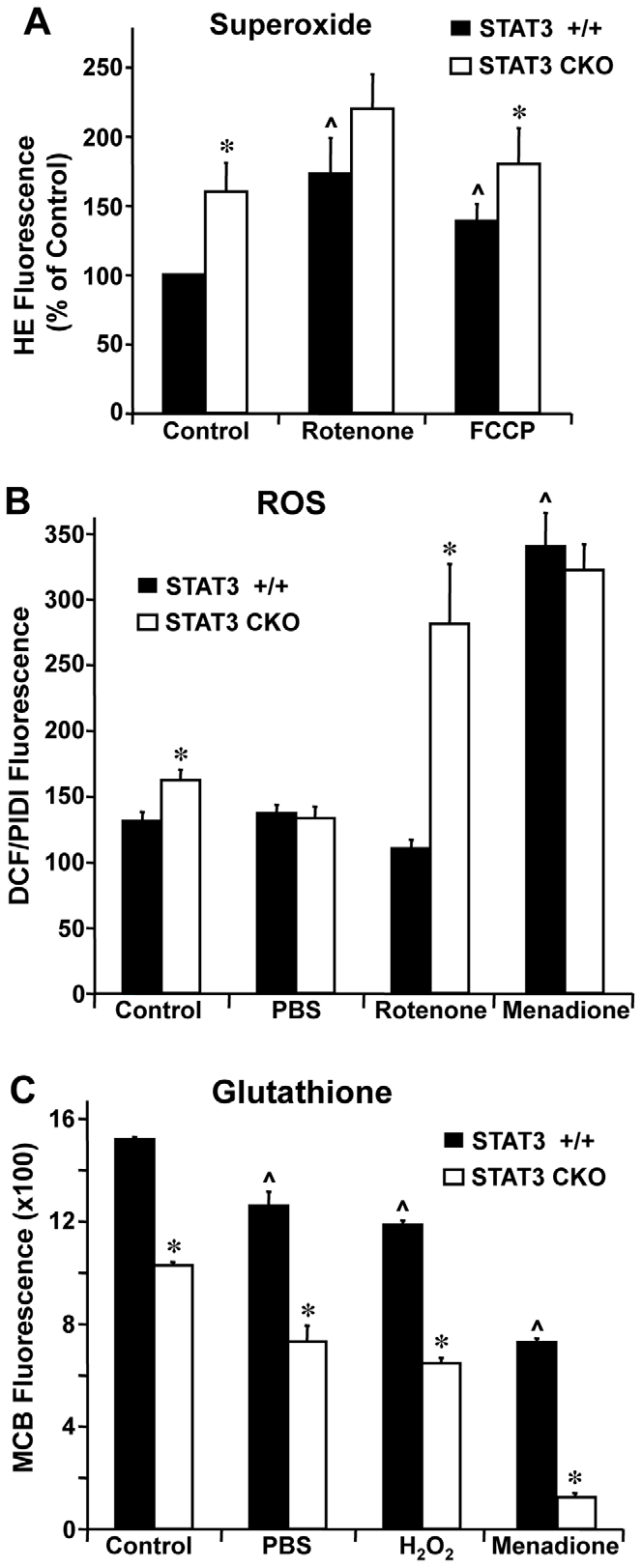
Oxidative stress studies using fluorescent probes. Cortical astrocytes cultured at passage 2 or 3 were assayed in the presence of 25 µM rotenone, 25 µM FCCP, 100 µM menadione, PBS, or 0.5% DMSO (vehicle control). (**A**) Superoxide generation was measured using the fluorescent probe HE as described in Materials and Methods and was consistently higher in STAT3-CKO cells. Data, expressed as per cent of STAT3 +/+ control, represent means of 7–8 determinations ± SEM. * p<0.05 compared with STAT3 +/+. (**B**) Generation of ROS was measured using DCF. Values are expressed as DCF fluorescence after 1 hr incubation normalized to total cell number derived by PI fluorescence in the presence of 160 µM digitonin (PIDI). Values represent means of 6–34 determinations ± SEM. * p<0.01 compared with STAT3 +/+ cells and ∧ p<0.05 compared with corresponding vehicle-treated control by 2-way ANOVA with Bonferroni post-hoc test. (**C**) Glutathione (GSH) levels were measured using 40 µM MCB after 30 min incubation at 37 °. Relative fluorescence values were normalized to total cell number and represent means of 6 determinations ± SEM. * p<0.001 compared with corresponding STAT-3 +/+ cells and ∧ p<0.05 compared with corresponding control using 2-way ANOVA with Bonferroni post-hoc test.

We next compared astrocyte properties contributing to oxidative stress and antioxidant defense. Superoxide (O_2_^−^) levels were assessed using the fluorescent probe, HE. Superoxide generation over a 30-minute period was 56% greater in STAT3-CKO astrocytes relative to STAT3 +/+ astrocytes (p<0.02) under control culture conditions (Fig. 2A). The complex I inhibitor, rotenone, significantly increased superoxide production in both STAT3 +/+ and STAT3-CKO astrocytes, but did so to a lesser extent in STAT3-CKO astrocytes as compared to STAT3 +/+ astrocytes. Similar results were obtained with the mitochondrial uncoupler, FCCP.

ROS were measured using DCF. This probe fluoresces on reaction with H_2_O_2_, hydroxyl radical, nitiric oxide and peroxynitrite, but does not react with superoxide [23]. ROS levels were significantly higher by 15% in STAT3-CKO astrocytes relative to STAT3 +/+ astrocytes (p<0.05) under control culture conditions (Fig. 2B). Rotenone significantly and markedly increased ROS by 73% in STAT3-CKO astrocytes relative to values under control conditions (p<0.001) and had no significant effect on ROS in STAT3 +/+ astrocytes. Menadione, a generator of ROS via redox cycling [24], [25], significantly and markedly increased ROS by over 90% in both STAT3 +/+ and STAT3-CKO astrocytes relative to values under control conditions (p<0.001) and eliminated the significant difference between the two cell types. Glucose starvation by transfer of cells from control culture conditions to PBS eliminated the increased ROS generation by STAT3-CKO astrocytes relative to STAT3 +/+ astrocytes during a one-hour incubation.

Glutathione (GSH) is a major component of cellular antioxidant defense. To assess GSH levels, we used the fluorescent probe, MCB. GSH levels were significantly lower by 30% in STAT3-CKO astrocytes relative to STAT3 +/+ astrocytes (Fig. 2A). We next compared GSH levels after subjecting astrocytes to glucose starvation or different forms of oxidative stress. Glucose starvation and H_2_O_2_ both moderately reduced GSH levels by about 20–35% in both STAT3 +/+ and STAT3-CKO astrocytes relative to control conditions, while retaining the significant reductions in STAT3-CKO astrocytes as compared to STAT3 +/+ astrocytes. Menadione significantly and markedly decreased GSH by 50% in STAT-3 +/+ astrocytes, and by 90% in STAT3-CKO astrocytes (p<0.001) relative to control culture conditions.

### Absence of STAT3 Impairs Astrocyte Mitochondrial Function and ATP Production

**Figure 3:**
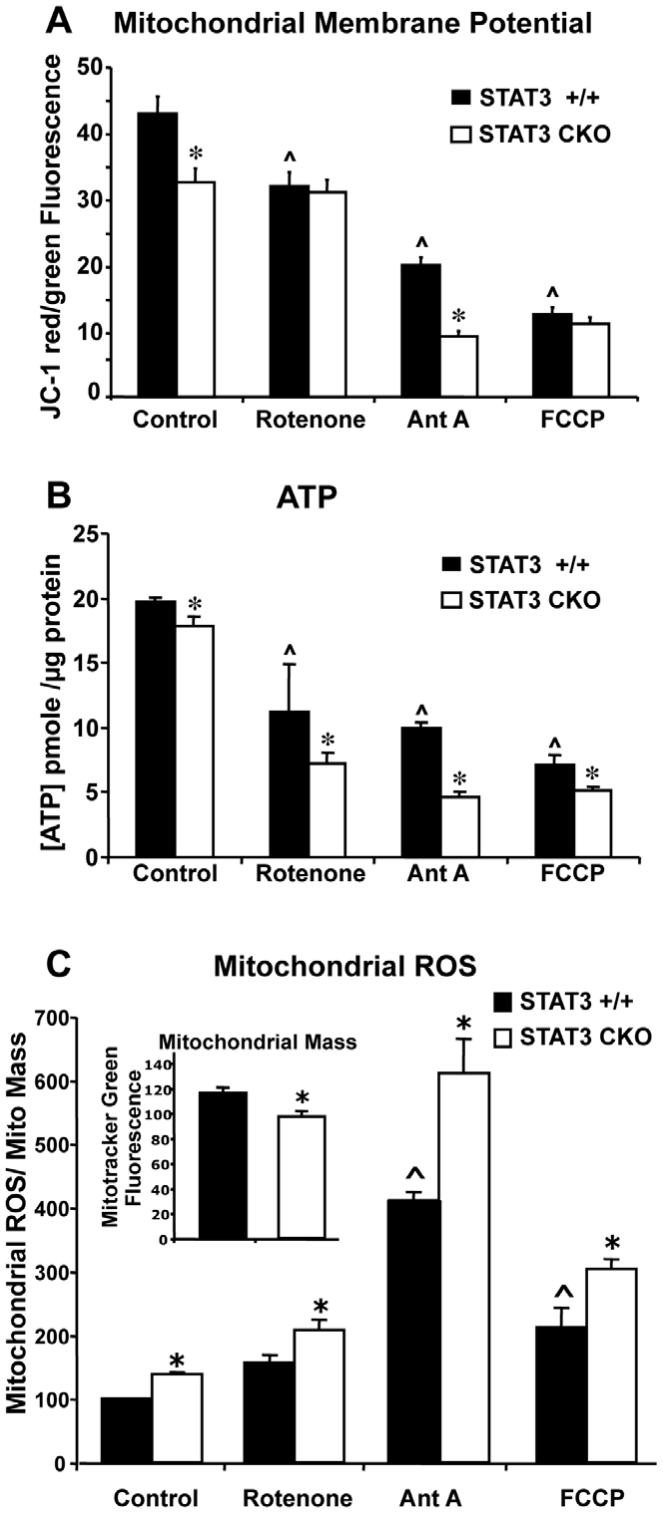
Analysis of mitochondrial function. Mitochondrial membrane potential (**A**), ATP levels (**B**), and mitochondrial ROS (**C**) of cortical astrocytes. Cells treated with the mitochondrial inhibitors 25 µM rotenone, 10 µM antimycin A (Ant A)or 25 µM FCCP. (**A**) Cells cultured in 48-well plates were first exposed to the inhibitors or vehicle control (0.5% DMSO) for 1 hr prior to addition of 1µM JC-1. After an additional hour of incubation in a CO_2_ incubator, both red (Ex = 530, Em = 590 nm) and green (Ex = 485, Em = 530 nm) fluorescence was measured. After background subtraction, the ratio of red to green fluorescence was calculated as a measure of mitochondrial membrane potential. Values represent means of 16 determinations ± SEM. * p<0.05 compared with STAT3 +/+ using 2-way ANOVA with Bonferroni post-hoc test. ∧ p<0.05 compared with control. (**B**) ATP levels were measured using the Promega CellTitre-Glo luminescence assay and a standard curve for ATP quantification. Values represent means of 6 determinations ± SEM. The experiment was repeated twice with similar results. (**C**) Mitochondrial-specific ROS assays were performed in 96-well culture plates as described in Materials and Methods. Values represent means of 60 determinations ± SEM. * p<0.05 compared with STAT3 +/+ using 2-way ANOVA with Bonferroni post-hoc test. ∧ p<0.05 compared with vehicle-treated control cells.

We next looked for potential effects of STAT3-CKO on astrocyte cell energetics. Mitochondrial membrane potential was assessed with the dual wavelength fluorescent probe, JC-1. The ratio of red/green JC-1 fluorescence was significantly lower by 25% in STAT3-CKO astrocytes relative to STAT3 +/+ astrocytes (p<0.001) under control culture conditions (Fig. 3A). We then compared the effects of inhibitors of mitochondrial functions. Rotenone, a selective inhibitor of complex I, decreased mitochondrial membrane potential in STAT3 +/+ astrocytes but not in STAT3-CKO astrocytes, thereby eliminating the significant difference between the two cell types. Antimycin A, a selective inhibitor of complex III, significantly decreased mitochondrial membrane potential in both STAT3 +/+ and STAT3-CKO astrocytes, while retaining a significant relative reduction in STAT3-CKO astrocytes as compared to STAT3 +/+ astrocytes. FCCP, a mitochondrial uncoupler, significantly and markedly decreased mitochondrial membrane potential in both STAT3 +/+ and STAT3-CKO astrocytes by over 75%, and eliminated the significant difference between the two cell types.

We also assessed ATP levels which were significantly lower by 10% in STAT3-CKO astrocytes relative to STAT3 +/+ astrocytes (p<0.05) under control culture conditions (Fig. 3B). ATP levels were significantly decreased to varying degrees by rotenone, antimycin A and FCCP in both STAT3 +/+ and STAT3-CKO astrocytes, while in all cases retaining a significant relative reduction in STAT3-CKO astrocytes as compared to STAT3 +/+ astrocytes.

In order to determine if mitochondria contributed to the elevated ROS observed in STAT3-CKO cells, the fluorescent probe MitoSOX Red was used. Unlike DCF and hydroethidine, MitoSOX Red selectively fluoresces in and is retained by mitochondria as a function of ROS generation [26], [27]. For these studies, parallel measurements using Mitotracker Green were utilized to quantify total mitochondrial mass, as this probe selectively stains mitochondria independent of mitochondrial membrane potential or ROS generation [28]. While apparent mitochondrial mass was ∼15% lower in STAT3-CKO astrocytes compared with STAT3 +/+ (see inset Fig 3C), mitochondrial ROS was ∼35% higher in STAT3-CKO when normalized to MitoTracker Green fluorescence (Fig. 3C). The increased mitochondrial ROS in STAT3-CKO cells was also observed in the presence of mitochondrial inhibitors rotenone, FCCP, and antimycin A. The relative difference between STAT3 +/+ and STAT3-CKO was potentiated to a greater extent by antimycin A and FCCP than by rotenone.

### Cell Proliferation Is Decreased in STAT3-CKO Astrocyte Cultures

**Figure 4:**
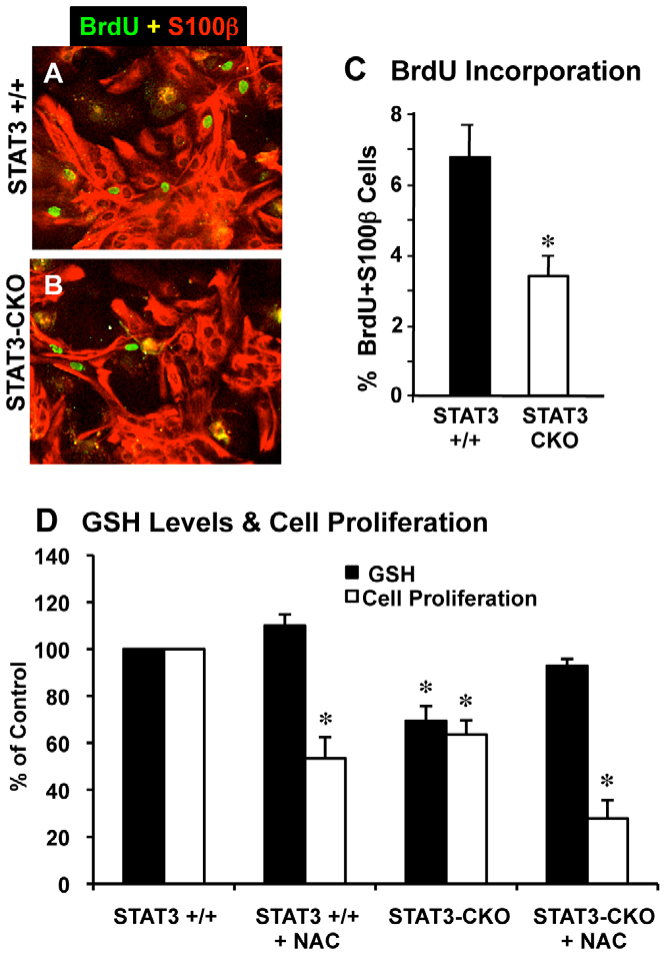
Astrocyte cell proliferation analyzed by BrdU incorporation and propidium iodide fluorescence. Merged images of double labeling immunofluorescence for S100β and BrdU (**A,B**) and graph (**C**) of cell counts show that significantly fewer S100β expressing astrocytes are dividing and labeled with BrdU in STAT3-CKO (**B**) compared with littermate control (**A**) cultures (n = 3 per group, * p<0.01 t-test). (**D**) Cells were cultured to passage 2 over a period of 3 weeks and plated into 96-well plates at a density of 5×10^3^/well. 0.5 mM N-actetylcysteine (NAC) was added 1, 3, and 5 days after plating. Cell number and GSH assays were performed as described in Materials and Methods. * p<0.01 compared with STAT3 +/+ control using one-way ANOVA with Tukey's post-hoc test.

STAT3 signaling has been implicated in regulating cell proliferation [29], [30]. We looked for influences of STAT3-CKO on primary astrocyte proliferation in several ways. Qualitatively we noted that STAT3-CKO cells required longer to reach confluence after passage and plating, suggesting a slower rate of proliferation. To evaluate this observation quantitatively, we first compared the number of S100β-positive astrocytes that incorporated BrdU administered as a pulse and found that about 3.5% of STAT3-CKO astrocytes incorporated BrdU over a 6 hour pulse, which was significantly lower and roughly half of the 7% of STAT3 +/+ astrocytes that incorporated BrdU over the same time period (Fig. 4A–C).

Cell proliferation was also quantified using a propidium iodide fluorescence assay. Cell numbers which were similar one day after plating was ∼35% lower in STAT3-CKO cell cultures compared with STAT3 +/+ after 7 days (Fig. 4D). Since oxidative stress is known to suppress cell division, we sought to determine if the observed higher levels of ROS in STAT3-CKO cells played a role in the lower proliferation rate. To address this issue, the proliferation assay was repeated in the presence of the antioxidant, N-acetylcysteine. Inclusion of 0.5 mM N-acetylcysteine in the medium elevated cellular GSH levels by 10% in STAT3 +/+ cells and by 34% in STAT3-CKO cells, producing levels of GSH similar to that in STAST3 +/+ cells. Despite these increases in GSH level, proliferation rate was not increased in either cell type. Similar results were obtained with 0.25 mM deferroxamine mesylate, and iron-chelating anti-oxidant (data not shown).

### Effects of Pharmacologic Inhibition of STAT3 Activation *In Vitro* Using AG490

**Figure 5:**
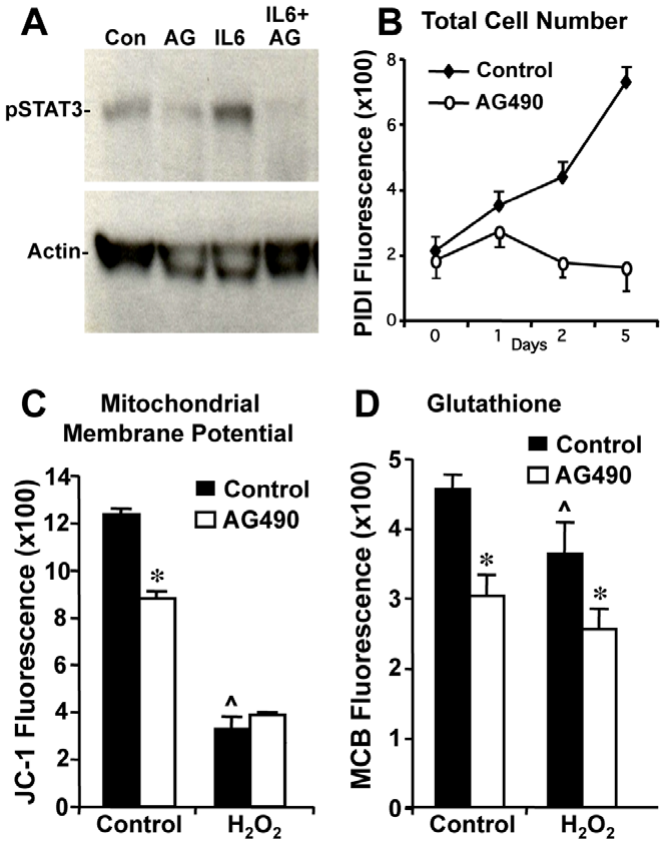
Effect of AG490 on cortical astrocyte cell function and STAT-3 phosphorylation. Cells from wild-type black C6 mice were pretreated for 2 hr with 25 µM AG490 followed by 1 hr exposure to 10 ng/ml interleukin 6 (IL-6) or 100 µm H_2_O_2_. (**A**) AG490 (AG) suppressed both basal and IL-6-stimulated STAT-3 phosphorylation. 25 µg of total protein was applied to each lane. (**B**) Prolonged AG490 exposure suppressed cell proliferation and reduced cell number after 1 day *in vitro*. Cell number was assessed by relative fluorescence of propidium iodide in the presence of 160 µM digitonin (**C, D**) Measurement of mitochondrial membrane potential using JC-1 and reduced GSH using MCB. Values represent means of 4 determinations ± SEM. These studies were repeated twice with similar results. * p<0.05 comparing AG490-treated with untreated cells. ∧ p<0.05 comparing H_2_O_2_- treated with corresponding control cells by 2-way ANOVA with Bonferroni post-hoc test.

We next compared the effects of STAT3-CKO with the pharmacological blockade of the STAT3 signaling pathway in astrocyte cultures prepared from wild-type mice using AG490, an inhibitor of Jak2 kinase. Dose response studies indicated that AG490 over a concentration range of 10 to 100 µM caused no increase in cell death measured with propidium iodide (data not shown). AG490 was used for subsequent studies at 25 µM, a concentration previously shown to prevent STAT3 tyrosine phosphorylation in vascular smooth muscle cells [31]. Treatment of wild-type astrocyte cultures with 25 µM AG490 suppressed STAT3 phosphorylation under basal conditions (with serum) as well as in response to added Il-6 (Fig. 5A). In addition, treatment of wild-type astrocyte cultures with AG490 reproduced changes in various cell functions observed in STAT3-CKO astrocytes. Astrocyte proliferation *in vitro* was significantly and markedly attenuated in wild-type astrocytes continuously exposed to AG490 (Fig. 5B). Mitochondrial membrane potential was significantly decreased by 30% in wild-type astrocytes exposed to AG490 for 2 hours (Fig. 5C). Exposure to the oxidative stress of H_2_O_2_ significantly and markedly reduced mitochondrial membrane potential in both control and AG490-treated astrocytes by over 60%, eliminating the significant difference between the two cell types (Fig. 5C). GSH levels were significantly lower by 35% in wild-type astrocytes exposed to AG490 for 2 hours (Fig. 5D). Exposure to H_2_O_2_ significantly reduced GSH levels by about 15% in both untreated and AG490-treated astrocytes relative to levels under control conditions, thereby retaining the significant reductions in AG490-treated astrocytes as compared to untreated astrocytes (Fig. 5D).

### Effects of STAT3-CKO on Astrocyte Gene Expression

To identify candidate molecules regulated or influenced by STAT3 signaling that might be involved with mechanisms related to mitochondrial function and the response to oxidative stress we studied global gene expression profiles using microarrays comparing astrocytes from STAT3-CKO mice with astrocytes from STAT3 +/+ mice. Cells were cultured to passage 3 over a period of 4–6 weeks and four biological replicates per condition were performed. Over 1200 genes exhibited statistically significant (p<0.005) increases or decreases in expression levels of 50% or more. As expected, and in agreement with Western data, GFAP mRNA was decreased nearly 5-fold in STAT3-CKO cells, as was the related intermediate filament nestin (Table 1).

**Table 1:** Gene expression differences: cytoskeletal and cell cycle proteins.

Abrev	Access. NM	Protein	CKO	(+/+)	CKO Effect	Fold Change	p value
GFAP	010277.1	Glial Fibrillary Acidic Protein	8.7^a^	11.0	−2.3	−4.9	0.0004
Nes	016701.2	Nestin	10.5	12.4	−1.9	−3.7	0.0011
CCND	007631.1	Cyclin D1	11.4	12.4	−1.0	−2.1	0.0023
Skp1A	001543.2	S-phase kinase-associated protein 1	11.3	11.8	−0.5	−1.4	0.0001
CDKN	009877.1	Cyclin dependant kinase inhibitor 2A	13.1	12.3	0.83	+ 1.8	0.028

a log2-transformed absolute RNA expression level.

As relates to the other experiments in this study, differences were observed in expression of genes involved cell cycle control and proliferation (Table 1), mitochondrial function (Table 2), oxidative stress and oxidative defense (Table 3), and apoptosis (Table 4). The majority of mitochondrial genes impacted were negatively regulated in the absence of STAT3 signaling, including NADH dehydrogenase NDUFS4 (1.4-fold decrease), MAP kinase 10 (2.1-fold decrease) and PARK7 (1.4-fold decrease) (Table 2). Notable among the gene expression differences were a number of genes involved in oxidative stress and antioxidant defense. STAT3-CKO cells displayed lower levels of mRNA for peroxiredoxin 5 (1.4-fold), peroxiredoxin 6 (2.1-fold), glutathione reductase (1.5-fold) and metallothione 2 (3.5-fold) compared with STAT3 +/+ cells (Table 3). Conversely glutathione synthetase, glutathione peroxidase 7, NADPH quinine oxidoreductase, and glutathione-S-transferase A3 mRNA were elevated (1.3-fold, 2.8-fold, 7.5-fold and 4.9-fold, respectively). mRNA for superoxide dismutases (SOD) 1 and 2 were not significantly different in STAT3-CKO cells. mRNA for SOD3 was 2-fold lower in STAT3-CKO cells although statistical significance was not quite attained n = 4 (p<0.11). Catalase levels were low and comparable in the two cell types. In addition several apoptosis-related genes were affected. Bcl-2 was reduced 2-fold while caspases 6 and 8 were elevated (50% and 30%, respectively) (Table 4).

**Table 2:** Gene expression differences: mitochondrial proteins.

Abrev	Access. NM	Protein	(CKO)	(+/+)	CKO Effect	Fold Change	p value
MAPK10	009158	Mitogen-activated Protein Kinase 10	7.3^a^	8.3	−1.09	−2.1	<0.00001
BACE2	019517.2	Beta-site API-cleaving enzyme 2	7.5	8.3	−0.8	−1.7	0.0009
PRDX5	012021.1	Peroxiredoxin 5	14.2	14.8	−0.61	−1.5	0.0002
PARK7	020569.1	Parkinson Disease 7	13.8	14.3	−0.49	−1.4	0.0033
NDUFS4	010887.1	NADH Dehydrogenase	13.5	13.9	−0.48	−1.4	0.0002
GPX7	024198.1	GPX 7	11.9	10.4	−1.5	+2.8	0.0026

a log2-transformed absolute RNA expression level.

**Table 3:** Gene expression differences: oxidative stress and defense proteins.

Abrev	Access. NM	Protein	(CKO)	(+/+)	CKO Effect	Fold Change	p value
Mt 2	008630.1	Metallothioneine 2	10.1^a^	11.9	−1.8	−3.5	0.0001
IDH2	173011.1	Isocitrate Dehydrogenase a (NADP+)	10.1	11.3	−1.2	−2.3	0.0001
OPLAH	153122.1	5-oxoprolinase (ATP Hydrolyzing)	8.6	9.8	−1.2	−2.3	0.003
PRDX6	007453.2	Peroxiredoxin 6	10.7	11.8	−1.1	−2.1	0.0077
SOD3	011435.2	Superoxide Dismutase 3	8.5	9.5	−1.0	−2.0	0.111
GPX4	008162	Glutathione Peroxidase 4	14.7	15.3	−0.6	−1.5	0.0003
GSR	010344.3	Glutathione Reductase	8.4	9.0	−0.6	−1.5	0.0011
PRDX5	012021.1	Peroxiredoxin 5	14.3	14.8	−0.5	−1.4	0.0002
NFE2L1	008686.2	Nuclear Factor -like 1	13.7	13.9	−0.2	−1.1	0.001
SOD2	013671.2	Superoxide Dismutase 2	12.0	12.1	−0.1	−1.1	0.208
SOD1	011434.1	Superoxide Dismutase 1	13.7	13.7	−0.0	1.0	0.533
GSS	008180.1	GSH Synthetase	10.1	9.7	+0.4	+1.3	0.085
GGTLA1	001820.2	γ-Glutamyltransferase-like Activity 1	7.9	7.2	+0.7	+1.6	0.0003
GPX 7	024198.1	GSH Peroxidase 7	11.9	10.4	+1.5	+2.8	0.0026
ANPEP	008486,1	Alanyl (Membrane) Aminopeptidase	9.8	8.3	+1.5	+2.8	0.0046
GSTA3	010356.2	Gl Glutathione S-transferase A3	11.4	9.1	+2.3	+4.9	<0.00001
Ptges	022415.2	Prostaglandin E Synthetase	10.2	7.6	+2.6	+6.1	<0.00001
NQO1	008706.1	NAD(P)H Dehydrogenase Quinone 1	12.1	9.2	+2.9	+7.5	<0.00001
Ptgis	008968.2	Prostagland I (prostacyclin) Synthetase	13.8	8.7	+5.1	+34.3	<0.00001

a log2-transformed absolute RNA expression level.

**Table 4:** Gene expression differences: apoptosis.

Abrev	Access. NM	Protein	(CKO)	(+/+)	CKO Effect	Fold Change	p value
BCL2	009741.2	B-cell CLL/Lymphoma 2	9.6^a^	10.3	−0.7	−1.6	<0.00001
TXNDC1	028338.1	Thioredoxin Domain Containing 1	9.4	9.8	−0.4	−1.3	0.0004
TNFRSf6	007987.1	Fas (TNF Receptor Superfamily, 6)	10.0	11.1	−1.1	−2.1	<0.00001
CASP8	009812.2	Caspase 8	11.0	10.5	0.5	+1.4	0.0361
CASP6	009811.2	Caspase 6	11.6	11.0	0.6	+1.5	0.0011
DAPK1	029653.1	Death-Associated Protein Kinase 1	9.1	8.4	0.7	+1.6	0.0001
TNFRSf22	023680.2	Fas (TNF Receptor Superfamily, 22)	12.1	10.6	1.5	+2.8	<0.00001

a log2-transformed absolute RNA expression level.

## Discussion

Previous studies with transgenic mice expressing a conditional deletion of the STAT-3 gene in astroglial cells demonstrated the role of STAT-3 in regulating astrogliosis [1]. While astrogliogenesis resulted in normal astrocyte numbers and morphology in these mice, cellular response to injury was significantly altered. In addition to GFAP synthesis, nestin and vimentin were underexpressed and cells demonstrated impaired ability to regulate inflammation. These findings raised the possibility that intrinsic properties of STAT3-CKO astrocytes diminished their capacity to withstand stress and their ability to protect neurons. In this study we sought to determine the effects of STAT3 deletion on astroglial mitochondrial functions and on oxidative stress response and defense.

One established function of astrocytes is to limit the oxidative stress of ROS generation resulting from the high rate of neuronal oxygen consumption. Astroglial protection from oxidative stress has been documented with excitotoxicity [32]–[34], neuropathological disorders (eg., Alzheimer's, Parkinson's, ALS) [35]–[37], autoimmune diseases (eg., multiple sclerosis) [38], [39], and heavy metal [40], [41] or chemical [42], [43] neurotoxicity. A common tool used for detection of ROS is the cell-permanent fluorescent probe DCF-DA which becomes oxidized and fluorogenic in the presence of H_2_O_2_, OH•, peroxinitrite and other oxidants [44]. Using this method ROS was 23% higher in STAT3-CKO cells compared with STAT3 +/+ cells. This pattern was not observed in the absence of glucose and was strongly enhanced in the presence of 25 µM rotenone, suggesting differential sensitivity of complex I of the electron transport chain in the two cell types. However, DCF-DA fails to detect the superoxide anion. Using the probe, HE, which specifically detects superoxide anion [45], we observed 66% higher basal levels of superoxide production in STAT3-CKO cells. Rotenone increased superoxide production two-fold but attenuated the relative difference between STAT3 +/+ and STAT3-CKO cells to 25%, further suggesting that STAT3 may ultimately impact complex I of the mitochondrial electron transport chain. This STAT3-dependent difference in superoxide generation was also diminished with the uncoupling agent, FCCP, suggesting a dependence on the mitochondrial membrane potential gradient. One possible explanation for the elevation and O^−^_2_ would be lower levels of superoxide dismutase in STAT3-CKO cells. STAT3 has been shown to up-regulate expression of MnSOD (SODII) in hepatocytes [46], cardiomyocytes [47], and hippocampal neurons [48]. In support of this hypothesis, gene expression array studies suggested that there may be small decreases in mRNA for SOD enzymes in the STAT3-CKO cells.

Elevated levels of NADPH quinone dehydrogenase (7.6-fold), GSH peroxidase 7 (2.5-fold) and GSH synthetase (30%) in STAT3-CKO cells are indicative of ARE promoter activation and chronic oxidative stress. However, several other genes normally associated with the ARE pathway such as hemoxygenase I and peroxiredoxin 6 were not elevated. Decreased expression of peroxiredoxins, metallothionein 2, and GSH reductase may be contributing factors to lower levels of reduced GSH and increased oxidative stress.

Numerous indicators of mitochondrial function were diminished in STAT3-CKO cells. Apparent mitochondrial mass, as revealed by MitoTracker Green, was lower by ∼15%. Mitochondrial membrane potential and cellular ATP levels were also lower. These abnormalities suggested to us that mitochondria may play a contributing role to the elevation in ROS in STAT3-CKO cells. Evidence for this effect was provided by studies using MitoSOX Red. When normalized to mitochondrial mass, mitochondrial-specific ROS was 38% higher in STAT3-CKO cells. Antimycin A potentiated the difference between STAT3 +/+ and STAT3-CKO cells and produced an ∼6-fold greater stimulation of mitochondrial ROS than did rotenone.

Gene expression array studies also identified decreased expression of NDUFS4, a subunit of NADH dehydrogenase of complex I of the mitochondrial electron transport chain. Since complex I is a source of ROS generation, abnormal function of this site could account for both increased production of superoxide anion and diminished ATP production [49], [50]. Further evidence for a role of astrocyte STAT3 in mitochondrial function is provided by studies of cell energetics. Using JC-1, mitochondrial membrane potential was found to be 25% lower in STAT3-CKO cells compared with STAT3 +/+ cells. This mitochondrial effect was accompanied by a 10% decrease in cellular ATP. The dissimilarity in membrane potential values was eliminated by rotenone exposure, as this agent did not change mitochondrial membrane potential in STAT3-CKO cells while lowering it by ∼25% in STAT3 +/+ cells. Antimycin A, however, caused a 70% reduction in mitochondrial membrane potential in STAT3-CKO cells and had a more potent effect on ATP level and mitochondrial ROS generation relative to rotenone. These findings again suggested that the STAT3 effect on mitochondria was dependent on mitochondrial complex I function. Recent studies have identified a direct interaction between STAT3 and mitochondria in cells from heart and liver [51], [52]. This interaction appeared to be dependent on serine phosphorylation of STAT3 and independent of transcriptional activity of the STAT3 protein. Mitochondrial respiration was reduced via inhibition of activities of complexes I and II of the electron transport chain. Our present observations of increased superoxide generation and decreased mitochondrial function modified by rotenone in STAT3-CKO cells support this novel mechanism of STAT3 action in cortical astrocytes.

Results from studies using the Jak-2 kinase inhibitor, AG490, with enriched cortical astrocyte cell cultures confirmed the notion that prevention of STAT3 activation alters cellular function and compromises cell defense capabilities. Two-hr exposure to 25 µM AG490 suppressed basal and Il-6-induced STAT3 phosphorylation and lowered astrocyte GSH levels, mitochondrial membrane potential and rate of cell proliferation. The magnitudes of these effects were similar to those observed when comparing astrocytes from STAT3-CKO and control STAT3 +/+ mice. Unlike the STAT3-CKO model, wherein STAT3 activation was absent selectively in the astrocyte lineage throughout pre- and postnatal development, AG490-treated cells were acutely deprived of activated STAT3 for only two hr after many days of normal activity. Our results suggest that the duration of STAT3 inhibition in normal, unstressed cells was of little consequence for the effects on mitochondrial and cellular defense properties examined in this study.

The present studies identify several abnormalities in astrocytes lacking the STAT3 gene. Mitochondria displayed lower mass and were less efficient in maintaining their membrane potential and producing ATP. Elevated rates of superoxide generation in STAT3-CKO cells and loss of GSH are indicative of oxidative stress. Finally, lower rates of DNA synthesis and cell proliferation were observed. The decreased cell proliferation was not corrected by antioxidants and likely resulted from altered expression of cell cycle control and apoptosis regulatory genes. These defects would likely compromise the ability of astrocytes to promote gliosis and to protect neurons [53].
